# Spin‐Decoupled Interference Metasurfaces for Complete Complex‐Vectorial‐Field Control and Five‐Channel Imaging

**DOI:** 10.1002/advs.202204664

**Published:** 2022-10-26

**Authors:** Tong Wu, Quan Xu, Xueqian Zhang, Yuehong Xu, Xieyu Chen, Xi Feng, Li Niu, Fan Huang, Jiaguang Han, Weili Zhang

**Affiliations:** ^1^ Center for Terahertz Waves and College of Precision Instrument and Optoelectronics Engineering the Key Laboratory of Optoelectronics Information and Technology (Ministry of Education) Tianjin University 300072 Tianjin P. R. China; ^2^ Guangxi Key Laboratory of Optoelectronic Information Processing School of Optoelectronic Engineering Guilin University of Electronic Technology Guilin 541004 P. R. China; ^3^ School of Electrical and Computer Engineering Oklahoma State University Stillwater OK 74078 USA

**Keywords:** complete complex‐vectorial‐field control, encryption, hologram, metasurfaces, terahertz

## Abstract

Light is a complex vectorial field characterized by its amplitude, phase, and polarization properties, which can be further represented by four basic parameters, that is, amplitudes and phases of two orthogonally polarized components. Controlling these parameters simultaneously and independently at will using metasurfaces are essential in arbitrarily manipulating the light propagation. However, most of the studies so far commonly require a great number of different meta‐atoms or rely on diffraction under oblique incidence, which lack convenience and flexibility in design and implementation. Here, a new metasurface paradigm is proposed that can completely manipulate the amplitudes and phases of two spin components based on the interference effect, where only two different meta‐atoms are applied. For proof‐of‐concept demonstration, two five‐channel meta‐holograms for imaging and information encryption are designed and experimentally characterized. The interference method provides a simple route toward compact complex and multifunctional meta‐devices.

## Graphical abstract

A new designing method for achieving complete complex‐vectorial‐field control is proposed based on spin‐decoupled interference metasurface through controlling the Pancharatnam–Berry phases of only two pairs of meta‐atoms with phase‐retarded half‐waveplate feature. As proof‐of‐concept demonstrations, two meta‐holograms which can achieve five‐channel imaging and information encryption are experimentally realized. The method provides a new avenue toward versatile novel and multi‐functional polarization devices.

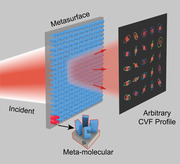

## 1 Introduction

Generally, coherent light can be described by two orthogonally polarized components of certain amplitudes and phases.^[^ ^1^ ^]^ Completely controlling all these four parameters is the key to achieve arbitrary manipulation over the complex vectorial field (CVF) of light, including amplitude, phase, and polarization distributions. During the last decades, metasurfaces composed of artificially two‐dimensional subwavelength meta‐atoms have attracted a wide range of attention due to their unprecedented ability in tailoring the light field at will.^[^ ^2^, ^3^, ^4^, ^5^ ^]^ Metasurfaces have led to many exciting electromagnetic phenomena and novel devices, such as beam deflectors,^[^ ^6^, ^7^, ^8^, ^9^ ^]^ meta‐lenses,^[^ ^10^, ^11^, ^12^, ^13^ ^]^ special beam generators,^[^ ^14^, ^15^, ^16^, ^17^ ^]^ and meta‐holograms.^[^ ^18^, ^19^, ^20^, ^21^, ^22^, ^23^ ^]^ However, majority of the studies can only control three parameters at most owing to the limited number of effective controlling degrees of freedom, such as the phase of one certain polarized component,^[^ ^6^, ^7^, ^11^ ^]^ the phases of two orthogonally polarized components,^[^ ^24^, ^25^ ^]^ the amplitude and phase of one certain polarized component,^[^ ^26^, ^27^ ^]^ and the phases of two orthogonally polarized components and their amplitude ratio.^[^ ^28^ ^]^

Recently, interference metasurfaces have emerged as an effective route to achieve complete control over these four parameters.^[^ ^29^, ^30^, ^31^, ^32^, ^33^ ^]^ The design strategy is to introduce multiple phase control meta‐atoms into one cell, that is, a meta‐molecule. The mechanism behind is to transfer two phase control degrees of freedom of two meta‐atoms into one amplitude and one phase control degrees of freedom of the meta‐molecule through interference. Thus, complete phase control in two orthogonal polarization channels in meta‐atom level becomes the key of this strategy. For the linear polarization basis, dynamic phase is quite applicable. However, it requires a great number of anisotropic meta‐atoms of different sizes to construct the complete interference meta‐molecule database.^[^ ^32^, ^33^ ^]^ Meanwhile, the control is discrete, which leads to controlling errors. For circular polarization basis, Pancharatnam–Berry (PB) phase is preferred, but it has to combine with other phase control methods to decoupled the responses of the two spin states, such as detour phase,^[^ ^29^, ^30^, ^31^ ^]^ and dynamic phase.^[^ ^32^ ^]^ The introduction of detour phase, however, requires the meta‐molecule period to be larger than the working wavelength to allow enough room for meta‐atom movements. In addition, it needs oblique incidence to efficiently control the diffraction beam. The introduction of dynamic phase encounters the same complication as that of the linear polarization. As for the other orthogonal polarization basis, they also have the problems described above.^[^ ^32^ ^]^

**Figure 1:**
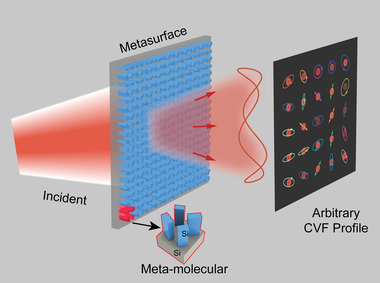
Schematic of the proposed metasurface for achieving complete CVF control. The proposed single‐layer dielectric metasurface can achieve independent and simultaneous control over the amplitudes and phases of the LCP and RCP transmitted waves based on spin‐decoupled interference effect. By designing the superposition of the field distributions of the transmitted LCP and RCP components, the amplitude, phase, and polarization of the overall transmitted field can be arbitrarily controlled.

In this article, we propose a novel and straightforward interference paradigm using single‐layer transmission‐type dielectric metasurface for achieving complete spin‐decoupled amplitude and phase control in the terahertz regime. We show that only two pairs of meta‐atoms with phase‐shifted half‐waveplate (HWP) feature are enough to construct the meta‐molecule database for analytically achieving the complete control by simply tuning their PB phases in a good accuracy. In principle, as long as the incident light contains both the left‐handed circularly polarized (LCP) and right‐handed circularly polarized (RCP) components, it is possible to generate arbitrarily relative amplitude, phase, and polarization distributions by designing the superposition of the output RCP and LCP fields, as schematically illustrated in **Figure** **1**. This is experimentally demonstrated by two functional devices, that is, a five‐channel imaging meta‐hologram and an encryption meta‐hologram constructed by binary spot array. Our method provides a simple way to achieve complete light control and may find broad applications in polarization optics.

## 2 Results

### 2.1 Design Principle

As mentioned above, there are total four independent parameters that need to be tuned, which thus require at least four independent controlling degrees of freedom. To give a clear explanation of the proposed interference method, we begin with the interference between four identical HWP meta‐atoms with independent rotation angles. In circular polarization basis, the corresponding transmission matrix *T*_c_ can be expressed as:

(1) $$\begin{array}{ccc} & & T_{c}=\left[\begin{array}{cc} t_{\mathrm{LL}} & t_{\mathrm{LR}}\\ t_{\mathrm{RL}} & t_{\mathrm{RR}} \end{array}\right]\\ & & \;=\frac{1}{4}\left[\begin{array}{cc} 0 & e^{-i2\theta_{1}}+e^{-i2\theta_{2}}+e^{-i2\theta_{3}}+e^{-i2\theta_{4}}\\ e^{i2\theta_{1}}+e^{i2\theta_{2}}+e^{i2\theta_{3}}+e^{i2\theta_{4}} & 0 \end{array}\right] \end{array}$$

where the former and latter subscripts of *t* represent the transmitted and incident polarization states with L and R depicting LCP and RCP, *θ*_1_ to *θ*_4_ represent the rotation angles of the four meta‐atoms, ±2*θ*_1_ to ±2*θ*_4_ in the exponents represent the corresponding PB phases, respectively. It is seen that the interference effect does make the amplitudes and phases of *t*_LR_ and *t*_RL_ independently tunable. However, their responses always conjugate with each other owing to the nature of the PB phase method. This is also true when the number of rotating meta‐atoms increases. Additional controlling factor is thus required to decouple this relation.^[^ ^34^ ^]^ Here, we find that introducing an overall *π*/2 relative phase shift to two of the above four meta‐atoms are just enough, where the corresponding transmission matrix *T*_c_ is rewritten as:

(2) $$\;T_{c}=\left[\begin{array}{cc} t_{\mathrm{LL}} & t_{\mathrm{LR}}\\ t_{\mathrm{RL}} & t_{\mathrm{RR}} \end{array}\right]=\frac{1}{4}\left[\begin{array}{cc} 0 & e^{-i2\theta_{1}}+e^{-i2\theta_{2}}+ie^{-i2\theta_{3}}+ie^{-i2\theta_{4}}\\ e^{i2\theta_{1}}+e^{i2\theta_{2}}+ie^{i2\theta_{3}}+ie^{i2\theta_{4}} & 0 \end{array}\right]$$

Clearly, the simple introduction of a complex coefficient *i* breaks the conjugate relation of the interference in *t*_LR_ and *t*_RL_ and makes the complete control by *θ*_1_ to *θ*_4_ possible. We suppose that the incident polarization in circular polarization basis is ${\left[A_{L}^{\mathrm{in}},A_{L}^{\mathrm{in}}e^{i\delta^{\mathrm{in}}}\right]}^{T}$ where $A_{L}^{\mathrm{in}}$ and $A_{R}^{\mathrm{in}}$ are the amplitudes of the LCP and RCP components with

(3) $${\left(A_{L}^{\mathrm{in}}\right)}^{2}+{\left(A_{R}^{\mathrm{in}}\right)}^{2}=1$$

and *δ^in^* is their phase difference, whereas the target amplitudes and phases of the transmitted LCP and RCP components are *A*_L_, *A*_R_ and *φ*_L_, *φ*_R_, respectively. Their relation can be analytically built by controlling *θ*_1_ to *θ*_4_ through the following equations (see Note S1, Supporting Information):

(4a) $$\;\theta_{1}=f_{1}\left(A_{L}e^{i\varphi_{L}},A_{R}e^{i\varphi_{R}}\right)=\frac{\mathrm{angle}\left[{\left(\frac{A_{L}e^{i\varphi_{L}}}{A_{R}^{\mathrm{in}}e^{i\delta^{\mathrm{in}}}}\right)}^{*}+\frac{A_{R}e^{i\varphi_{R}}}{A_{L}^{\mathrm{in}}}\right]+\cos^{-1}\left\{\mathrm{abs}\left[{\left(\frac{A_{L}e^{i\varphi_{L}}}{A_{R}^{\mathrm{in}}e^{i\delta^{\mathrm{in}}}}\right)}^{*}+\frac{A_{R}e^{i\varphi_{R}}}{A_{L}^{\mathrm{in}}}\right]\right\}}{2}$$

(4b) $$\theta_{2}=f_{2}\left(A_{L}e^{i\varphi_{L}},A_{R}e^{i\varphi_{R}}\right)=\frac{\mathrm{angle}\left[{\left(\frac{A_{L}e^{i\varphi_{L}}}{A_{R}^{\mathrm{in}}e^{i\delta^{\mathrm{in}}}}\right)}^{*}+\frac{A_{R}e^{i\varphi_{R}}}{A_{L}^{\mathrm{in}}}\right]-\cos^{-1}\left\{\mathrm{abs}\left[{\left(\frac{A_{L}e^{i\varphi_{L}}}{A_{R}^{\mathrm{in}}e^{i\delta^{\mathrm{in}}}}\right)}^{*}+\frac{A_{R}e^{i\varphi_{R}}}{A_{L}^{\mathrm{in}}}\right]\right\}}{2}$$

(4c) $$\theta_{3}=f_{3}\left(A_{L}e^{i\varphi_{L}},A_{R}e^{i\varphi_{R}}\right)=\frac{\frac{\pi }{2}+\mathrm{angle}\left[{\left(\frac{A_{L}e^{i\varphi_{L}}}{A_{R}^{\mathrm{in}}e^{i\delta^{\mathrm{in}}}}\right)}^{*}-\frac{A_{R}e^{i\varphi_{R}}}{A_{L}^{\mathrm{in}}}\right]+\mathrm{co}s^{-1}\left\{\mathrm{abs}\left[{\left(\frac{A_{L}e^{i\varphi_{L}}}{A_{R}^{\mathrm{in}}e^{i\delta^{\mathrm{in}}}}\right)}^{*}-\frac{A_{R}e^{i\varphi_{R}}}{A_{L}^{\mathrm{in}}}\right]\right\}}{2}$$

(4d) $$\theta_{4}=f_{4}\left(A_{L}e^{i\varphi_{L}},A_{R}e^{i\varphi_{R}}\right)=\frac{\frac{\pi }{2}+\mathrm{angle}\left[{\left(\frac{A_{L}e^{i\varphi_{L}}}{A_{R}^{\mathrm{in}}e^{i\delta^{\mathrm{in}}}}\right)}^{*}-\frac{A_{R}e^{i\varphi_{R}}}{A_{L}^{\mathrm{in}}}\right]-\cos^{-1}\left\{\mathrm{abs}\left[{\left(\frac{A_{L}e^{i\varphi_{L}}}{A_{R}^{\mathrm{in}}e^{i\delta^{\mathrm{in}}}}\right)}^{*}-\frac{A_{R}e^{i\varphi_{R}}}{A_{L}^{\mathrm{in}}}\right]\right\}}{2}$$

It is noticed that, the complete spin‐decoupled amplitude and phase control here requires that the largest amplitude of the overall output must be less than

(5) $$A_{max}=A_{L}^{\mathrm{in}}\times A_{R}^{\mathrm{in}}/\left(A_{L}^{\mathrm{in}}+A_{R}^{\mathrm{in}}\right)$$

to get feasible solutions (real numbers) of *θ*_1_ to *θ*_4_ (see Note S1, Supporting Information). Beyond this range, the *A*_L_‐*φ*_L_‐*A*_R_‐*φ*_R_ space is not fully filled. But this does not affect its real application, since the complete CVF control is determined by the relative relation among the meta‐molecules’ transmission responses. In terms of only polarization control without considering the overall amplitude and phase, arbitrary point on the Poincare sphere can be theoretically generated using this method as long as the incident light contains both the LCP and RCP components (see Note S2 and Figure S1, Supporting Information).

### 2.2 Meta‐Molecule Design and Selection

**Figure 2:**
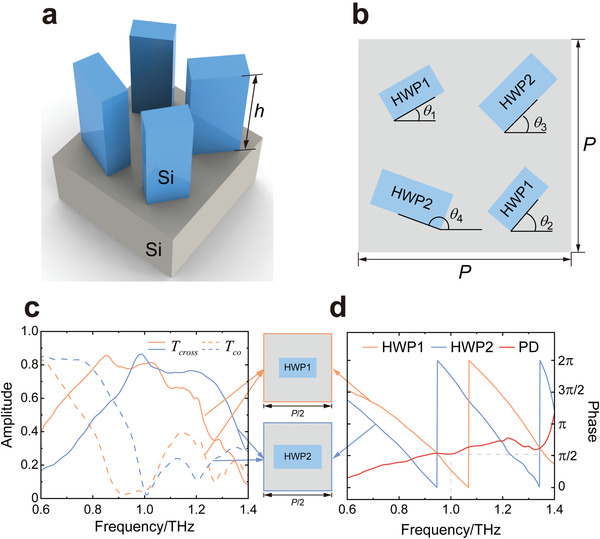
Design of the spin‐decoupled interference meta‐molecule. a) 3D and b) top views of the designed meta‐molecule which is composed by two pairs of meta‐atoms made of silicon pillars with transvers dimensions (widths, lengths) of (41 µm, 77 µm) and (49 µm, 99 µm), height of *h* = 200 µm and period of *P* = 280 µm. Simulated c) cross‐ and co‐polarized transmission amplitude spectra and d) cross‐polarized transmission phase spectra of the two meta‐atoms under circularly polarized incidences. The red curve in (d) represents the cross‐polarized phase difference spectrum between the two meta‐atoms. The insets between (c) and (d) show the schematics of the two meta‐atoms in simulations.

To verify the above design principle, a dielectric meta‐molecule composed by two pairs of rectangular‐shape silicon‐pillar meta‐atoms are designed, as schematically illustrated in **Figure** **2**a,b. The meta‐molecule is arranged in a square lattice of period *P* = 280 µm. The two pairs of meta‐atoms are placed at the centers of the four quadrants of the lattice. Their heights are all *h* = 200 µm, while their transverse dimensions (widths, lengths) are (41 µm, 77 µm) and (49 µm, 99 µm), respectively. Figure 2c,d illustrate the simulated cross‐ and co‐polarized transmission amplitude spectra and cross‐polarized transmission phase spectra of the two meta‐atoms in a period of *P*/2 = 140 µm under circularly polarized incidences. At the designed frequency of 1.0 THz, there are nearly no co‐polarized transmissions while the cross‐polarized transmission amplitudes approach ≈0.82 (see Figure 2c), whereas there is a *π*/2 phase difference between the two cross‐polarized transmission phases (see the red curve and gray dashed lines in Figure 2d). Such features satisfy the above‐derived conditions very well.

**Figure 3:**
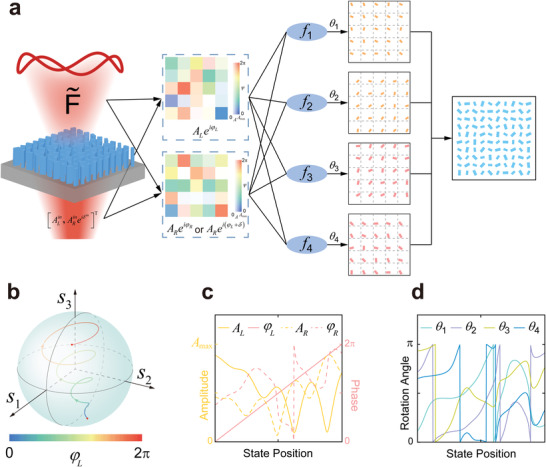
Principle of the metasurface design. a) A general design flow of metasurface for achieving complete amplitude, phase and polarization control. b) Schematic of a Poincare sphere with radius of $A_{\max}^{2}$. The inset color line represent the CVF to be generated. c) The extracted amplitude and phase distributions of the LCP and RCP components of the CVF along the color line in (b). d) Calculated rotation angle distributions of the four meta‐atoms corresponding to (c).

A general approach to achieve arbitrarily desired amplitude and phase distributions of the transmitted LCP and RCP components using the proposed meta‐molecules is schematically illustrated in **Figure** **3**a. First, decomposing the desired interfacial CVF distribution into LCP and RCP component field distributions, including their amplitude and phase distributions. Second, scaling the amplitude distributions of both LCP and RCP component fields into a range of 0 to *A*_max_ according to the desired incident polarization. Then, calculating the orientation angle distributions of the four meta‐atoms point by point respectively using Equation (4). At last, putting the four distributions of rotated meta‐atoms together to obtain the metasurface. Equation (4) also reveals that the desired rotation angles of *θ*_1_ to *θ*_4_ are determined by not only the desired field distributions but also the incident polarization. The performance will be influenced when the incident polarization deviates from the design.

Figure 3b–d give a numerical example of this method under *x*‐polarized incidence. Figure 3b illustrates a polarization Poincare sphere with *s*_1_ to *s*_3_ being the Stokes parameters and the radius being $A_{\max}^{2}$ of the database. The inset spiral color line represents an arbitrarily desired vectorial field distribution to be generated, where the modulus of the coordinate of each point on the line represents its field amplitude, the corresponding normalized coordinate represents its polarization state, and the color represents the phase of its LCP component. Figure 3c illustrates the extracted amplitude and phase distributions of the LCP and RCP components from the line in Figure 3b. The corresponding rotation angle distributions of the four meta‐atoms calculated to achieve such fields are illustrated in Figure 3d.

### 2.3 Five‐Channel Imaging Meta‐Hologram

**Figure 4:**
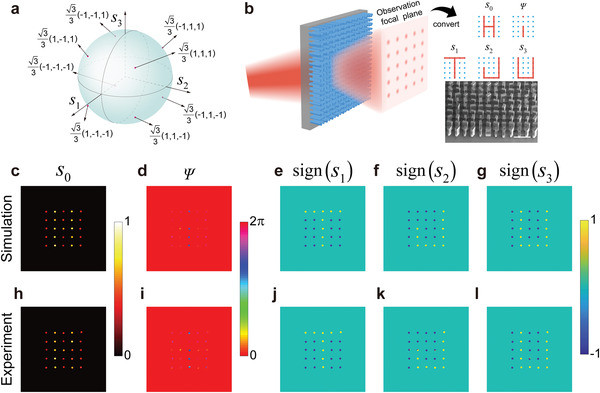
Five‐channel imaging meta‐hologram. a) Eight selected polarization states on a standard Poincare sphere to be generated in the spot array image. b) Schematic of the function of the five‐channel imaging meta‐hologram. The inset shows the tilted SEM image of part of the fabricated meta‐hologram. Scale bar: 300 µm. c–g) Extracted spot array images in the *s*_0_, *Ψ*, *s*_1_, *s*_2_, and *s*_3_ channels from the simulated CVF distribution at the imaging plane at 1.0 THz. Only the corresponding values around the spot positions are presented. For the images in the *s*_1_, *s*_2_, and *s*_3_ channels, they are calculated by taking sign function of the extracted values. Clearly images of five letters “Hi TJU” are obtained. h–l) Corresponding measured results to (c–g) at 1.05 THz. The incident polarization is *x*‐polarized.

As we mentioned above that light of given frequency has three basic properties: Amplitude, phase, and polarization, where the polarization can be further symbolized using three Stokes parameters. Here, the complete CVF control is divided into five channels, which can be described by *s*_0_, *Ψ*, *s*_1_, *s*_2_, and *s*_3_ with

(6) $$s_{0}=\sqrt{s_{1}^{2}+s_{2}^{2}+s_{3}^{2}}$$

representing the CVF intensity and *Ψ* representing the phase of the LCP component, respectively. For simplifying the meta‐hologram design, the images are set as 5 × 5 spot array in a square lattice. Meanwhile, the values of the five channels at these spots are all binarized to show contrast images. For *s*_0_, the binary values after normalization are 0.5 and 1, representing dark and bright spots. For *Ψ*, a phase difference of *π*/3 is set between the phases of the dark and bright spots. For *s*_1_, *s*_2_, and *s*_3_, the binary values after separate normalization are all $+\sqrt{3}/3$ and $-\sqrt{3}/3$. Thus, there are totally 8 polarizations states located at the centers of the 8 quadrants of the standard Poincare sphere, as illustrated in **Figure** **4**a.

Based on the configuration given above, a five‐channel imaging meta‐hologram is designed, which can separately show holographic spot images of letters “H,” “i,” “T,” “J,” “U” in the five channels, as schematically illustrated in Figure 4b. By extracting the actual CVF information in each spot, the corresponding amplitudes and phases of the LCP and RCP components can be calculated. To generate such a spot array, here we adopt the design scheme of flat meta‐lens by considering each spot as an independent focal, thus the required CVF distribution of the meta‐hologram can be obtained by superposition of those of the meta‐lenses as:

(7) $$\begin{array}{c} \left[\begin{array}{c} A_{L}e^{i\varphi_{L}}\\ A_{R}e^{i\varphi_{R}} \end{array}\right]\propto \left[\begin{array}{c} \sum_{\mathrm{mn}}A_{L}^{\mathrm{mn}}\exp\left[i\frac{2\pi }{\lambda }\left(f-\sqrt{x_{\mathrm{mn}}^{2}+y_{\mathrm{mn}}^{2}+f^{2}}\right)+i\psi_{\mathrm{mn}}\right]\\ \sum_{\mathrm{mn}}A_{R}^{\mathrm{mn}}\exp\left[i\frac{2\pi }{\lambda }\left(f-\sqrt{x_{\mathrm{mn}}^{2}+y_{\mathrm{mn}}^{2}+f^{2}}\right)+i\delta_{\mathrm{mn}}\right.\\ \left.+\;i\psi_{\mathrm{mn}}\right] \end{array}\right] \end{array}$$

where *m* and *n* represent the serial number of the focal in the 5 × 5 spot array with *m, n* ∈ {1, 2, 3, 4, 5}, *f* represents the focal length of the meta‐lenses and also the distance between meta‐hologram and the spot array image which is set as 40 mm, *λ* represents the wavelength which is 300 µm, $A_{L}^{\mathrm{mn}}$, $A_{R}^{\mathrm{mn}}$ are the desired amplitudes of the LCP and RCP components of the spot *mn* at the position (*x_mn_, y_mn_*) with *δ_mn_* being their phase difference and *ψ_mn_* being the phase of the LCP component.

With the calculated results using Equation (7), the meta‐hologram can be obtained based on the approach in Figure 3a. In the design, the lattice of the spot array is set as 2.1 mm, the incident light is set as *x*‐polarized and the working frequency is set at 1.0 THz. By discretizing the calculated CVF distribution in a range of 21 mm × 21 mm in a step of *P*, the final five‐channel meta‐hologram is obtained. To characterize its imaging performance, numerical simulation is first carried out. After extracting the CVF at 1.0 THz, the values of the variables in the five channels are calculated. The results are illustrated in Figure 4c–g. Only the values around a small area of the spots are presented, while the others are simply set as zero to eliminate the background disturbance. This numerical operation corresponds to placing a mask of circle array at the imaging plane in real cases.^[^ ^35^ ^]^ Here, to increase the imaging tolerance, the images in the *s*_1_, *s*_2_, and *s*_3_ channels are presented by taking the corresponding signs of the extracted values instead of presenting the actual normalized values. Detailed information on the CVF information processing can be found in Figure S2, Supporting Information. Clear images of five letters “Hi TJU” are obtained, showing the effectiveness of our method.

To characterize the imaging performance experimentally, the five‐channel meta‐hologram is fabricated using conventional photolithography and deep reactive ion etching method onto a high‐resistance silicon wafer, and measured using a fiber‐based near‐field scanning terahertz microscope system (see Section 4 and Figure S3, Supporting Information). The tilted scanning electron microscopy (SEM) image of part of the fabricated meta‐hologram is shown in the lower right corner of Figure 4b. Figure 4h–l illustrate the measured results in the five channels at 1.05 THz, which agree well with the simulations. The slight blue shift of the best‐performance frequency can be attributed to the dimensional deviation from the fabrication imperfections.

### 2.4 Information Encryption Meta‐Hologram

As described above, we demonstrated that our spin‐decoupled interference metasurface could be used to achieve five‐channel binary spot array images owing to its ability in complete CVF control. Though the spot array image is simple, it can be used to store encrypted information. For each spot in our case, it can in principle carry five bits of coding information. Here, we propose an encryption strategy based on such an imaging ability, in which the images in *Ψ* and *s*_0_ channels are set as two “key” images for decoding the information, and the images in *s*_1_, *s*_2_, and *s*_3_ channels are used to store encrypted information. To represent enough characters, the *s*_1_, *s*_2_, and *s*_3_ values of two adjacent spots are combined to encode a six‐digit binary number, that is, *u*( $s_{1}^{2i-1}$)*u*( $s_{2}^{2i-1}$)*u*( $s_{3}^{2i-1}$)*u*( $s_{1}^{2i}$)*u*( $s_{2}^{2i}$)*u*( $s_{3}^{2i}$) with *i* being an integer and the *u*(·) represents step function, which can thus represent 64 characters based on the pre‐customized cipher book, similar to the ASCII code.

**Figure 5:**
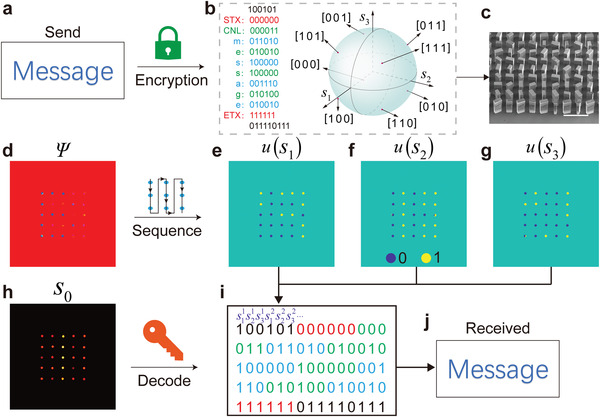
Information encryption meta‐hologram. a) The top secret content to be sent, which is a word “Message.” b) Compiling method of the word “Message” to 6‐digit binary codes using the *s*_1_, *s*_2_, and *s*_3_ of the selected eight polarization states. Each code is composed by the *s*_1_, *s*_2_, and *s*_3_ values of two adjacent spots. c) Tilted SEM image of part of the fabricated meta‐hologram. Scale bar: 300 µm. d) Measured image in the *Ψ* channel for providing the key information of reading sequence. e–g) Measured images in the *s*_1_, *s*_2_, and *s*_3_ channels after taking step function operations for providing the encrypted coding information. h) Measured image in the *s*_0_ channel for providing the key information of the cipher book number. i) Extracted 75 bits of binary codes from (e–g) based on the reading sequence guided by (d). j) Decoded secret information from (i) using the cipher book indexed by (h). The results are all measured at 1.05 THz under *x*‐polarized incidence.

The working and designing principle of the information encryption meta‐hologram is schematically illustrated in **Figure** **5**. It supposes that user A wants to send a top secret content “Message” to user B, as illustrated in Figure 5a. First, the content needs to be compiled to binary code based on a cipher book. For the 5 × 5 spot array, there are total 75 bits of binary codes in the *s*_1_, *s*_2_ and *s*_3_ channels representing the polarization states, where 42 bits represent the seven characters in the secret content, 18 bits represent three special characters including start of text (STX), capitalize the next letter, and end of text (ETX), and the residual 15 bits can be designed as random useless information for confusing the stealer. Detail compiling example is illustrated in Figure 5b. For the images in the *Ψ* and *s*_0_ channels, they can be respectively compiled to present certain symbols to indicate the reading sequence of the spots in the array and the index number of the cipher book. Second, based on the above compiling process, the corresponding CVF information at each spot is determined, which can then be used to design the information encryption meta‐hologram based on the same designing procedure of the five‐channel imaging meta‐hologram under the pre‐promised polarized incidence. Here, the incident wave is also set as *x*‐polarized at 1.0 THz in the design process. The corresponding tilted SEM image of part of the fabricated meta‐hologram is illustrated in Figure 5c.

Finally, when user B receives the meta‐hologram, he or she should use suitable terahertz equipment to measure the imaging performance. The corresponding measured images in the five channels are illustrated in Figure 5d–h. As the first step, the image in the *Ψ* channel shows a letter “B,” which can be endowed with the meaning of reading the *s*_1_, *s*_2_, and *s*_3_ information in a pre‐indexed sequence, as illustrated in the right inset of Figure 5d (see Figure S4, Supporting Information, for more exemplary custom reading sequences). Then, user B can transfer the measured images in the *s*_1_, *s*_2_, and *s*_3_ channels (see Figure 5e–g) to a string of 75 bits of binary codes by taking the step function *u*(∙) to them using the above reading sequence, as illustrated in Figure 5i. At last, to decode the encrypted information, user B has to check the measured image in the *s*_0_ channel which is number “1” indexing the number of the required cipher book (see Tables S1 and S2, Supporting Information). When finding the 6 digits codes of STX and ETX in the string in a step of 6 bits from the beginning, it means that the useful information is encrypted between them. Thus, user B can decode them to plaintext, as shown in Figure 5j. If we suppose that an evil person E steals the metasurface, besides the state‐of‐the‐art light beam information measuring, E also has to know the exact incident polarization, the effective working frequency range, details of the five channels, encryption method, rules of reading sequence, and the correct decoding cipher book, etc. Missing any of the information described above would frustrate the stealing behavior. Meanwhile, the compiling way, the channel functions and all of the above decryption and encryption rules can be customized in real time to further increase the confidentiality.

## 3 Discussion and Conclusion

In the above meta‐hologram designs, all the rotation angles are calculated based on ideal interference effect. However, there will be coupling effects among the meta‐atoms in real cases, especially when their spatial gap sizes are small, which will cause the transmission responses deviated from the model. To investigate this influence, a group of numerical simulations are carried out and compared with the calculations. The results show a strong correlation between them and the average deviation is 0.23, indicating a relative good consistence between the simulation and calculation (see Note S3 and Figure S5, Supporting Information). The comparison and the performance of the applied meta‐hologram strategy illustrate that the deviation induced by the coupling effect is in the applicable level. To reduce the coupling effect and increase the performance, one may compose the HWPs using highly localized resonators,^[^ ^36^, ^37^, ^38^ ^]^ which may help reduce the field overlap between the adjacent meta‐atoms.

The silicon pillar here can be regarded as an anisotropic truncated waveguide or effective medium, which relies on effective propagation phase difference to achieve HWP feature. Thus, the HWP bandwidth is narrow, as indicated by Figure 2c,d. Together with the coupling effect mentioned above, the working bandwidths of the meta‐holograms are further limited. To characterize the working bandwidth, the overall holographic performances at different frequencies are checked. For simulations, the working bandwidths of the five‐channel imaging and information encryption meta‐holograms are about 0.04 THz and 0.1 THz, respectively. For experiments, the values are both 0.02 THz. Beyond these bandwidths, the number of incorrect spots rises. Detailed results can be found in Figures S6–S9, Supporting Information. To increase the bandwidth, one can apply two broadband HWPs with similar dispersion, such as applying reflection‐type metasurfaces based on Mie or plasmonic resonances.^[^ ^39^, ^40^ ^]^

Besides, the efficiencies of the two meta‐holograms at different frequencies are also calculated by normalizing the integrated intensity over the filtered image (see Figure S2, Supporting Information) in the *s*_0_ channel to that over the transmitted field distribution from a bare silicon substrate. The simulated (measured) efficiencies of the five‐channel imaging and information encryption meta‐holograms are around 2.59% to 6.85% (0.85% to 2.03%), where the peak efficiencies both occur at 1.0 THz (1.05 THz) which are 6.39% and 6.85% (2.03% and 1.91%), respectively (see Figure S10, Supporting Information). The efficiencies are mainly limited by the applied complete CVF control here which has a maximum transmission amplitude of *A*_max_ = $\sqrt{2}/4$, and also affected by the actual transmission amplitudes of the meta‐atoms. The smaller efficiencies in the measured results can be attributed to the fabrication deviations.

As one focal spot can independently carry 5‐bit information, our method has much room to work with other encryption way for information transfer, such as the Baudot code. The information capacity is also possibly increased by dividing the *s*_0_ and *Ψ* channels into more levels, or introducing orbital‐angular‐momentum degree of freedom into each spot whose topological charge can serve as an additional bit. Furthermore, our method is universal which is compatible with mechanical technology to achieve dynamic complete control and associated meta‐devices, such as in the microwave regime where mechanical rotation in the unit‐cell level is possible.^[^ ^41^, ^42^ ^]^

In summary, we have proposed and demonstrated a generic designing strategy for achieving complete CVF control, including its amplitude, phase and polarization properties, based on spin‐decoupled interference metasurfaces controlled by the PB phase mechanisms in the terahertz regime. The basic meta‐molecule of the interference metasurface only contains two pairs of independently rotating meta‐atoms made of silicon pillars with HWP feature and *π*/2 overall relative phase shift. To show the capabilities of our method, we designed and experimentally demonstrated a five‐channel imaging meta‐hologram and an information encryption meta‐hologram. This design method would provide a new avenue toward versatile novel and multi‐functional optical devices.

## 4 Experimental Section

### Experimental Characterization

All the spots array was characterized by a self‐built fiber‐based near‐field scanning terahertz microscopy system, see Figure S3, Supporting Information. First, the *x*‐polarized terahertz waves generated from the emitter were collected and collimated by a lens of 50‐mm focus length. Then, a linear polarizer (P1) was used to further polarize the polarization to the *x* polarization. After passing through the meta‐holograms, another linear polarizer (P2) was used to allow analysis of the polarization of the transmitted complex field by measuring its 45°‐ and −45°‐polarized components through rotating P2 by either 45° or −45°, since they contributed the same polarization projection to the *x* polarization that the probe can detect. At last, the field distributions were mapped by two‐dimensionally scanning the meta‐holograms with the probe placed at 10 mm above them, where the scanning range was 20 mm × 20 mm and the scanning step was 0.2 mm.

The obtained terahertz fields at each scanning position were initially time‐domain signals, which were averaged from 15 measurements. By taking Fourier transform to them, the intensity and phase distributions of the 45°‐ and −45°‐polarized components at the desired frequency (1.05 THz) can be extracted. The single‐to‐noise ratio of the system at this frequency, defined as the ratio of the standard deviation (SD) to mean is 45.98. Notice that, the working plane designed here was 40 mm above the meta‐holograms. To obtain the corresponding field distributions, rigorous Rayleigh–Sommerfeld diffraction theory is applied

(8a) $$E_{+45,40\mathrm{mm}}\left(x,y\right)=\int \int E_{+45,10\mathrm{mm}}\left(x_{1},y_{1}\right)\frac{z\left(1-\mathrm{ikR}\right)e^{\mathrm{ikR}}}{2\pi R^{3}}dx_{1}{\mathrm{dy}}_{1}$$

(8b) $$E_{-45,40\mathrm{mm}}\left(x,y\right)=\int \int E_{-45,10\mathrm{mm}}\left(x_{1},y_{1}\right)\frac{z\left(1-\mathrm{ikR}\right)e^{\mathrm{ikR}}}{2\pi R^{3}}dx_{1}{\mathrm{dy}}_{1}$$

where *E*_+45,10 mm_, *E*_−45,10 mm_ and *E*_+45,40 mm_, *E*_−45,40 mm_, respectively, represent the complex fields of the 45°‐ and −45°‐polarized components at distances of 10 and 40 mm, *k* = 2*π*/*λ* is the wave vector, *z* = 30 mm is the field evolution space from the measuring plane (*x*_1_ *y*_1_ plane at 10 mm above the meta‐hologram) to the target plane (*xy* plane at 40 mm above the meta‐hologram), and

(9) $$R=\sqrt{{\left(x_{1}-x\right)}^{2}+{\left(y_{1}-y\right)}^{2}+z^{2}}$$

Then, the desired images in the five channels can be respectively calculated by

(10) $$\Psi =\mathrm{angle}\left[E_{+45,40\mathrm{mm}}+E_{-45,40\mathrm{mm}}+i\left(E_{+45,40\mathrm{mm}}-E_{-45,40\mathrm{mm}}\right)\right]$$

(11) $$s_{0}={\left|E_{+45,40\mathrm{mm}}\right|}^{2}+{\left|E_{-45,40\mathrm{mm}}\right|}^{2}$$

(12) s1=−2ReE+45,40mm*E−45,40mm

(13) $$s_{2}={\left|E_{+45,40\mathrm{mm}}\right|}^{2}-{\left|E_{-45,40\mathrm{mm}}\right|}^{2}$$

(14) s3=−2ImE+45,40mm*E−45,40mm

### Statistical Analysis

There are two sets of data presented in Figure S5, Supporting Information, showing the transmission responses of a group of meta‐molecules in circular polarization basis. They are respectively obtained by calculation using Equation (S3), Supporting Information, and simulation by finite difference time domain method. All the data are determined values. The sample size (*n*) for the statistical analysis was 1296. Excel was used for analyzing the data, in which average deviation and Pearson correlation were employed to analyze the relation between the two sets of data. Related information can be found in Note S3, Supporting Information. The measured results in Figures 4 and 5 are presented as mean distributions with a sample size (*n*) of 15 and a signal to noise ratio of 45.98 in each point.

## Conflict of Interest

The authors declare no conflict of interest.

## Supporting information

Supporting Information

## Data Availability

The data that support the findings of this study are available from the corresponding author upon reasonable request.
