# Supplementary material for: Spin‐Decoupled Interference Metasurfaces for Complete Complex‐Vectorial‐Field Control and Five‐Channel Imaging
Source: Adv Sci (Weinh). 2022 Oct 26;9(35):2204664. doi: 10.1002/advs.202204664 (PMC9762281; doi:10.1002/advs.202204664)
Supplement: Supplementary file 1 — Supporting Information [file ADVS-9-2204664-s001.pdf]

ADVANCED SCIENCE
Open Access

Supporting Information

for *Adv. Sci.*, DOI 10.1002/advs.202204664

Spin-Decoupled Interference Metasurfaces for Complete Complex-Vectorial-Field Control and Five-Channel Imaging

Tong Wu, Quan Xu, Xueqian Zhang, Yuehong Xu, Xieyu Chen, Xi Feng, Li Niu, Fan Huang, Jiaguang Han* and Weili Zhang**

Supporting Information

Spin-Decoupled Interference Metasurfaces for Complete Complex-Vectorial-Field Control and Five-Channel Imaging

Tong Wu, Quan Xu, Xueqian Zhang, Yuehong Xu, Xieyu Chen, Xi Feng, Li Niu, Fan Huang, Jiaguang Han* and Weili Zhang**

Note 1. Derivation of the complete CVF control

Equation (2) in the main text can be written to

$$T_c = \frac{1}{2}\begin{bmatrix} 0 & \cos(\theta_1-\theta_2)e^{-i(\theta_1+\theta_2)} + i\cos(\theta_3-\theta_4)e^{-i(\theta_3+\theta_4)} \\ \cos(\theta_1-\theta_2)e^{i(\theta_1+\theta_2)} + i\cos(\theta_3-\theta_4)e^{i(\theta_3+\theta_4)} & 0 \end{bmatrix}. \quad \text{(S1)}$$

Then, we have

$$\frac{1}{2}\begin{bmatrix} 0 & \cos(\theta_1-\theta_2)e^{-i(\theta_1+\theta_2)} + i\cos(\theta_3-\theta_4)e^{-i(\theta_3+\theta_4)} \\ \cos(\theta_1-\theta_2)e^{i(\theta_1+\theta_2)} + i\cos(\theta_3-\theta_4)e^{i(\theta_3+\theta_4)} & 0 \end{bmatrix}\begin{bmatrix} A_L^{in} \\ A_R^{in}e^{i\delta^{in}} \end{bmatrix} = \begin{bmatrix} A_L e^{i\varphi_L} \\ A_R e^{i\varphi_R} \end{bmatrix}. \quad \text{(S2)}$$

in which,

$$\frac{1}{2}\left[\cos(\theta_1-\theta_2)e^{-i(\theta_1+\theta_2)} + i\cos(\theta_3-\theta_4)e^{-i(\theta_3+\theta_4)}\right]A_R^{in}e^{i\delta^{in}} = A_L e^{i\varphi_L}, \quad \text{(S3a)}$$

$$\frac{1}{2}\left[\cos(\theta_1-\theta_2)e^{i(\theta_1+\theta_2)} + i\cos(\theta_3-\theta_4)e^{i(\theta_3+\theta_4)}\right]A_L^{in} = A_R e^{i\varphi_R}. \quad \text{(S3b)}$$

Afterward, by solving the above equations, we can get

$$\cos(\theta_1-\theta_2)e^{i(\theta_1+\theta_2)} = \left(\frac{A_L e^{i\varphi_L}}{A_R^{in}e^{i\delta^{in}}}\right)^* + \left(\frac{A_R e^{i\varphi_R}}{A_L^{in}}\right), \quad \text{(S4a)}$$

$$\cos(\theta_3-\theta_4)e^{i(\theta_3+\theta_4)} = i\left(\frac{A_L e^{i\varphi_L}}{A_R^{in}e^{i\delta^{in}}}\right)^* - i\left(\frac{A_R e^{i\varphi_R}}{A_L^{in}}\right). \quad \text{(S4b)}$$

It is clear from Equation (S4) that

$$\theta_1-\theta_2 = \cos^{-1}\left\{\text{abs}\left[\left(\frac{A_L e^{i\varphi_L}}{A_R^{in}e^{i\delta^{in}}}\right)^* + \frac{A_R e^{i\varphi_R}}{A_L^{in}}\right]\right\}, \quad \text{(S5a)}$$

$$\theta_1+\theta_2 = \text{angle}\left[\left(\frac{A_L e^{i\varphi_L}}{A_R^{in}e^{i\delta^{in}}}\right)^* + \frac{A_R e^{i\varphi_R}}{A_L^{in}}\right], \quad \text{(S5b)}$$

$$\theta_3 - \theta_4 = \cos^{-1}\left\{ \mathrm{abs}\left[\left(\frac{A_L e^{i\varphi_L}}{A_R^{in} e^{i\delta^{in}}} \right)^* - \frac{A_R e^{i\varphi_R}}{A_L^{in}} \right] \right\}, \tag{S5c}$$

$$\theta_3 + \theta_4 = \frac{\pi}{2} + \mathrm{angle}\left[\left(\frac{A_L e^{i\varphi_L}}{A_R^{in} e^{i\delta^{in}}} \right)^* - \frac{A_R e^{i\varphi_R}}{A_L^{in}} \right]. \tag{S5d}$$

At last, the four rotation angles θ_1 to θ_4 can be easily obtained

$$\theta_1 = \frac{\mathrm{angle}\left[\left(\frac{A_L e^{i\varphi_L}}{A_R^{in} e^{i\delta^{in}}} \right)^* + \frac{A_R e^{i\varphi_R}}{A_L^{in}} \right] + \cos^{-1}\left\{ \mathrm{abs}\left[\left(\frac{A_L e^{i\varphi_L}}{A_R^{in} e^{i\delta^{in}}} \right)^* + \frac{A_R e^{i\varphi_R}}{A_L^{in}} \right] \right\}}{2}, \tag{S6a}$$

$$\theta_2 = \frac{\mathrm{angle}\left[\left(\frac{A_L e^{i\varphi_L}}{A_R^{in} e^{i\delta^{in}}} \right)^* + \frac{A_R e^{i\varphi_R}}{A_L^{in}} \right] - \cos^{-1}\left\{ \mathrm{abs}\left[\left(\frac{A_L e^{i\varphi_L}}{A_R^{in} e^{i\delta^{in}}} \right)^* + \frac{A_R e^{i\varphi_R}}{A_L^{in}} \right] \right\}}{2}, \tag{S6b}$$

$$\theta_3 = \frac{\frac{\pi}{2} + \mathrm{angle}\left[\left(\frac{A_L e^{i\varphi_L}}{A_R^{in} e^{i\delta^{in}}} \right)^* - \frac{A_R e^{i\varphi_R}}{A_L^{in}} \right] + \cos^{-1}\left\{ \mathrm{abs}\left[\left(\frac{A_L e^{i\varphi_L}}{A_R^{in} e^{i\delta^{in}}} \right)^* - \frac{A_R e^{i\varphi_R}}{A_L^{in}} \right] \right\}}{2}, \tag{S6c}$$

$$\theta_4 = \frac{\frac{\pi}{2} + \mathrm{angle}\left[\left(\frac{A_L e^{i\varphi_L}}{A_R^{in} e^{i\delta^{in}}} \right)^* - \frac{A_R e^{i\varphi_R}}{A_L^{in}} \right] - \cos^{-1}\left\{ \mathrm{abs}\left[\left(\frac{A_L e^{i\varphi_L}}{A_R^{in} e^{i\delta^{in}}} \right)^* - \frac{A_R e^{i\varphi_R}}{A_L^{in}} \right] \right\}}{2}. \tag{S6d}$$

Noticed that, the target amplitudes and phases of the transmitted LCP and RCP components need to satisfy the following conditions to get real-number solutions of θ_1 to θ_4,

$$\mathrm{abs}\left[\left(\frac{A_L e^{i\varphi_L}}{A_R^{in} e^{i\delta^{in}}} \right)^* + \frac{A_R e^{i\varphi_R}}{A_L^{in}} \right] \leqslant 1, \tag{S7a}$$

$$\mathrm{abs}\left[\left(\frac{A_L e^{i\varphi_L}}{A_R^{in} e^{i\delta^{in}}} \right)^* - \frac{A_R e^{i\varphi_R}}{A_L^{in}} \right] \leqslant 1, \tag{S7b}$$

At the same time, the left terms of the above two equations should also satisfy

$$\mathrm{abs}\left[\left(\frac{A_L e^{i\varphi_L}}{A_R^{in} e^{i\delta^{in}}} \right)^* + \frac{A_R e^{i\varphi_R}}{A_L^{in}} \right] \leqslant \mathrm{abs}\left[\left(\frac{A_L e^{i\varphi_L}}{A_R^{in} e^{i\delta^{in}}} \right)^* \right] + \mathrm{abs}\left(\frac{A_R e^{i\varphi_R}}{A_L^{in}} \right) = \frac{A_L}{A_R^{in}} + \frac{A_R}{A_L^{in}}, \tag{S8a}$$

$$\mathrm{abs}\left[\left(\frac{A_L e^{i\varphi_L}}{A_R^{in} e^{i\delta^{in}}} \right)^* - \frac{A_R e^{i\varphi_R}}{A_L^{in}} \right] \leqslant \mathrm{abs}\left[\left(\frac{A_L e^{i\varphi_L}}{A_R^{in} e^{i\delta^{in}}} \right)^* \right] + \mathrm{abs}\left(\frac{A_R e^{i\varphi_R}}{A_L^{in}} \right) = \frac{A_L}{A_R^{in}} + \frac{A_R}{A_L^{in}}. \tag{S8b}$$

In order to have equal control over the output LCP and RCP components, a maximum

amplitude $A_{\max}$ is set so that their amplitudes are both no more than $A_{\max}$. By combing Equation (S7) and (S8), we have

$$\frac{A_{\max}}{A_R^{in}}+\frac{A_{\max}}{A_L^{in}}=1, \tag{S9}$$

where

$$A_{\max}=\frac{A_L^{in}A_R^{in}}{A_L^{in}+A_R^{in}}. \tag{S10}$$

The above derivation shows that the complete CVF control here has an upper bound of the output amplitude of $A_{\max}$. To use this complete database, the desired vectorial field distribution should be scaled into this range to get real-number solutions of θ_1 to θ_4.

Note 2. Complete polarization generation

To show the complete polarization generation ability of the proposed method, theoretical calculated polarization states are plotted in the Stokes parameter space to show the filling status. In the calculation, the four meta-atoms' rotation angles (θ_1 to θ_4) in one meta-molecule are raster varied from 0° to 170° with a 10° interval, and the incident polarization is set as *x* polarization ($\left[\sqrt{2}/2,\sqrt{2}/2\right]^{\mathrm{T}}$ in the circular polarization basis) as an example. **Figure S1**a shows the corresponding calculated polarization states using Equation (S3). It is seen that the polarization states can stack into a sphere with a radius of 0.5. In terms of only polarization state without considering its overall amplitude and phase, every point on the Poincare sphere can clearly be encircled. Theoretically, the proposed method can generate arbitrary polarization state as long as the incident light contains both the LCP and RCP components according to Equation (S3). The difference is the fact the generated polarization states will stack into an ellipsoid rather than a sphere when the incident polarization is not linearly polarized. Nonetheless, without considering the overall amplitude, a sphere can always be selected inside the ellipsoid.

It is worth pointing out that the complete CVF control here requires not only polarization state control but also its overall amplitude and phase control, or in other words, complete amplitude and phase control over the LCP and RCP outputs. This can only be fully achieved in a certain range that the amplitudes of the overall outputs are no more than $A_{\max}$, see Note 1. To visualize it, the polarization responses in the complete CVF range under *x*-polarized incidence are plotted in Figure S1b, as indicated by the red scatters. Owing to the complete

CVF control, the density in this range is very high. Each red scatter can represent a polarization state with an arbitrary overall phase. The maximum radii are 0.25 along s_1 and s_2 axes while 0.125 along s_3 axis.

Note 3. Coupling influence on the meta-molecule responses

To investigate this influence, a group of numerical simulations are carried out by raster varying the rotation angles of the four meta-atoms (θ_1 to θ_4) in the meta-molecule under x-polarized incidence, and compare the corresponding results at the working frequency of 1.0 THz with the calculated results using Equation (S3). The varying ranges are all from 0° to 150° with a 30° interval, so there are total 6^4 = 1296 meta-molecules. **Figure S5** shows the simulated (black lines) and calculated (red lines) results of all the meta-molecules in the forms of amplitudes and phases of the transmitted LCP and RCP components. It is seen that the overall simulated responses are consistent with the calculated responses. To quantitatively compare these two responses, the Pearson correlation coefficients between them are first calculated, which are 0.71, 0.53, 0.71 and 0.52 for the transmitted LCP amplitude A_L, RCP amplitude A_R, LCP phase φ_L and RCP phase φ_R, respectively. These suggest a strong correlation between the simulation and calculation.[S1] In addition, the deviation between the theoretical and simulated responses for each meta-molecule is also calculated, which is defined as $[|A_{Ls}\exp(i\varphi_{Ls}) - A_L\exp(i\varphi_L)|, |A_{Rs}\exp(i\varphi_{Rs}) - A_R\exp(i\varphi_R)|]_{\max}$ with the variables with and without the subscript s respectively representing the simulated and calculated responses. The average deviation of them is 0.23.

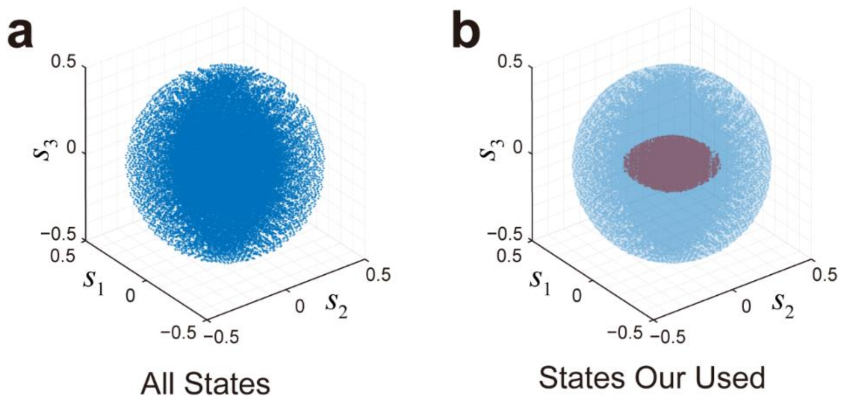

Figure S1. Calculated polarization distributions under *x*-polarized incidence. a) Polarization states (blue scatters) in Stokes parameter basis when θ_1 to θ_4 are raster varied from 0° to 170° with a 10° interval. b) Highlighted polarization states (red scatters) of the complete CVF control.

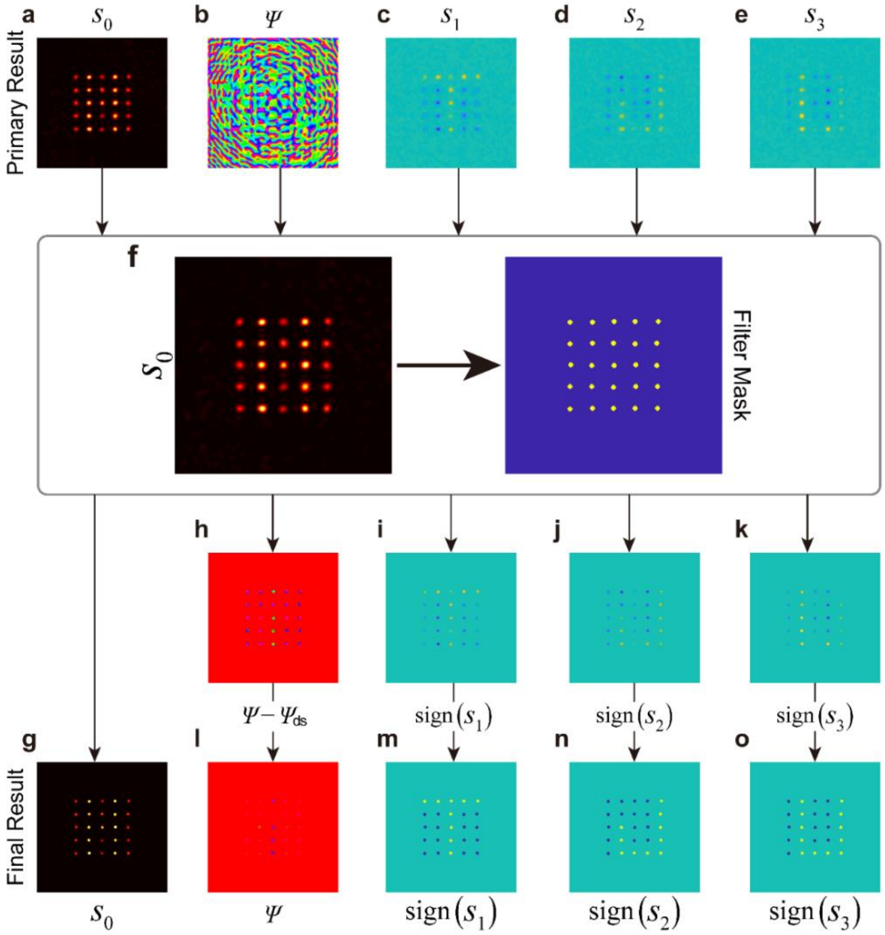

Figure S2. Numerical information processing on the holographic image. a-e) Simulated holographic images in the five channels. f) Generation of filter mask based on the image in s_0 channel. Each yellow spot in the filter mask circles a circular area centered at the maximum position of the corresponding spot in a) with 45-μm diameter. The values inside and outside the yellow spots are respectively set as 1 and 0. g-k) Calculated filtered images of a-e) by multiplying them with the generated filter mask. l-o) Post-processing results of h-k), where the image in Ψ channel is obtained by subtracting the phase of the dark spot Ψ_{ds} defined as the average phases of all the dark spots in g), and the images in s_1, s_2 and s_3 channels are obtained by taking sign function to i-k).

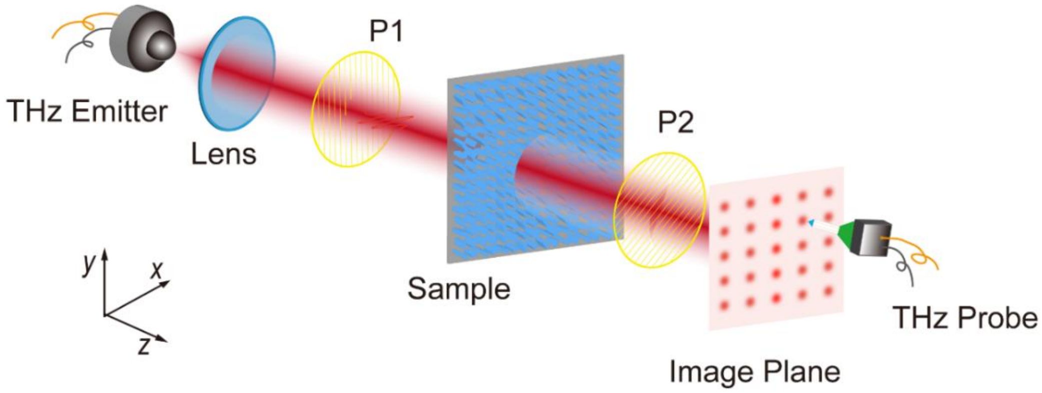

Figure S3. Schematic of the experimental setup. P1 and P2: metallic grid polarizers.

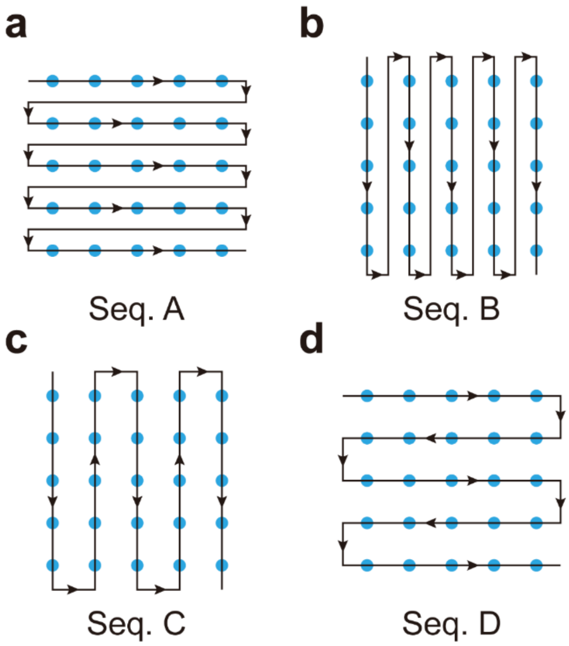

Figure S4. A series of exemplary custom reading sequences.

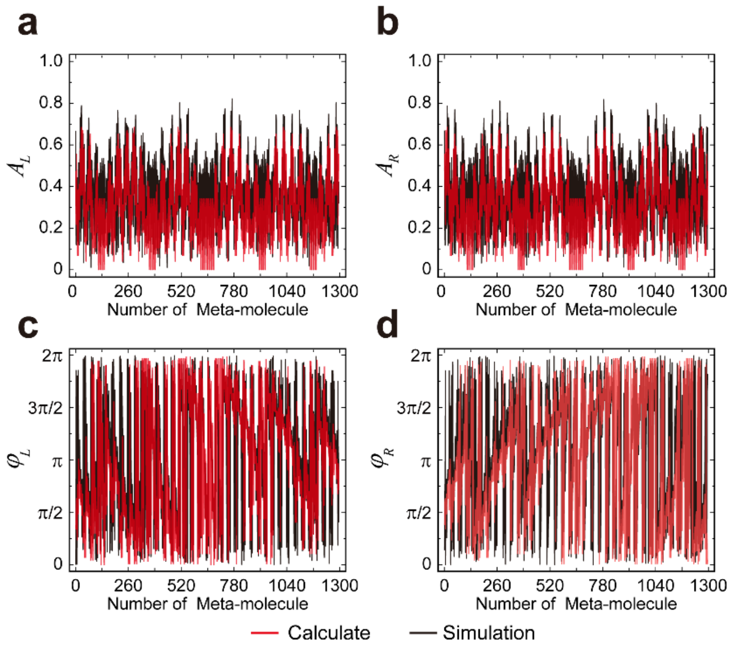

Figure S5. Comparison between simulated and calculated responses of meta-molecules with different θ_1 to θ_4. Simulated (black) and calculated (red) LCP and RCP transmission amplitudes a,b) and phases c,d) of different meta-molecules, respectively. The number of the horizontal axes are all from 1 to 1296. Each represents a combination of θ_1 to θ_4 whose varying ranges are all from 0° to 150° with a 30° interval.

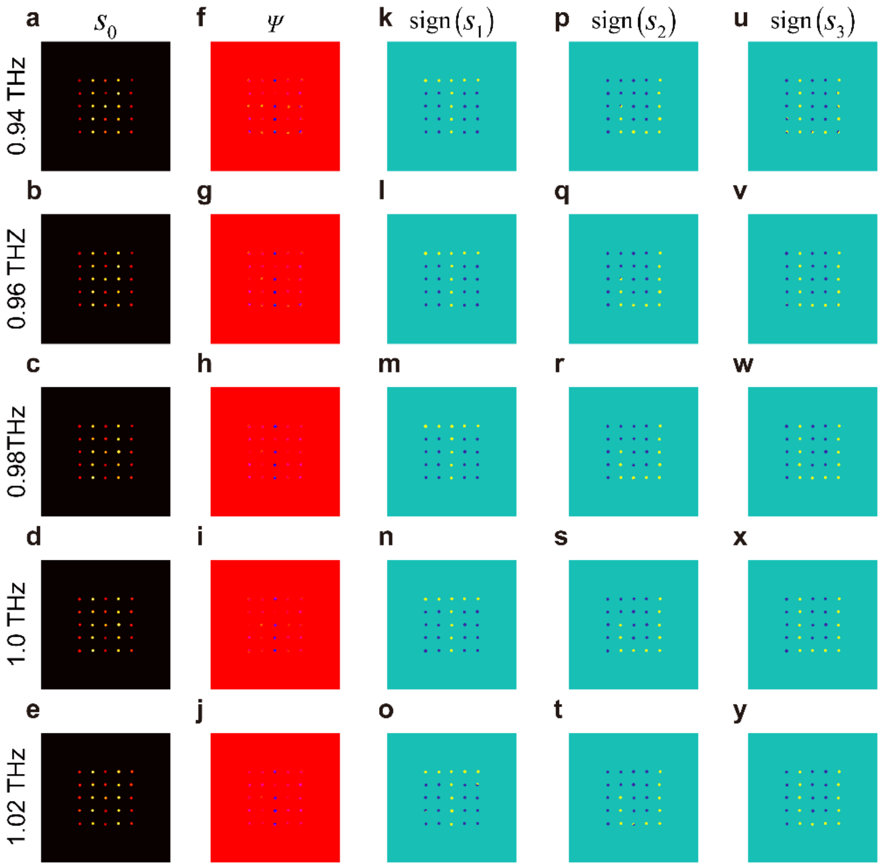

Figure S6. Simulation results of the five-channel imaging meta-hologram at different frequencies. Simulated distributions of the holographic image in the a-e) s_0, f-j) Ψ, k-o) s_1, p-t) s_2 and u-y) s_3 channels from 0.94 to 1.02 THz with a step of 0.02 THz, respectively. The working bandwidth is about 0.04 THz from 0.96 to 1.0 THz.

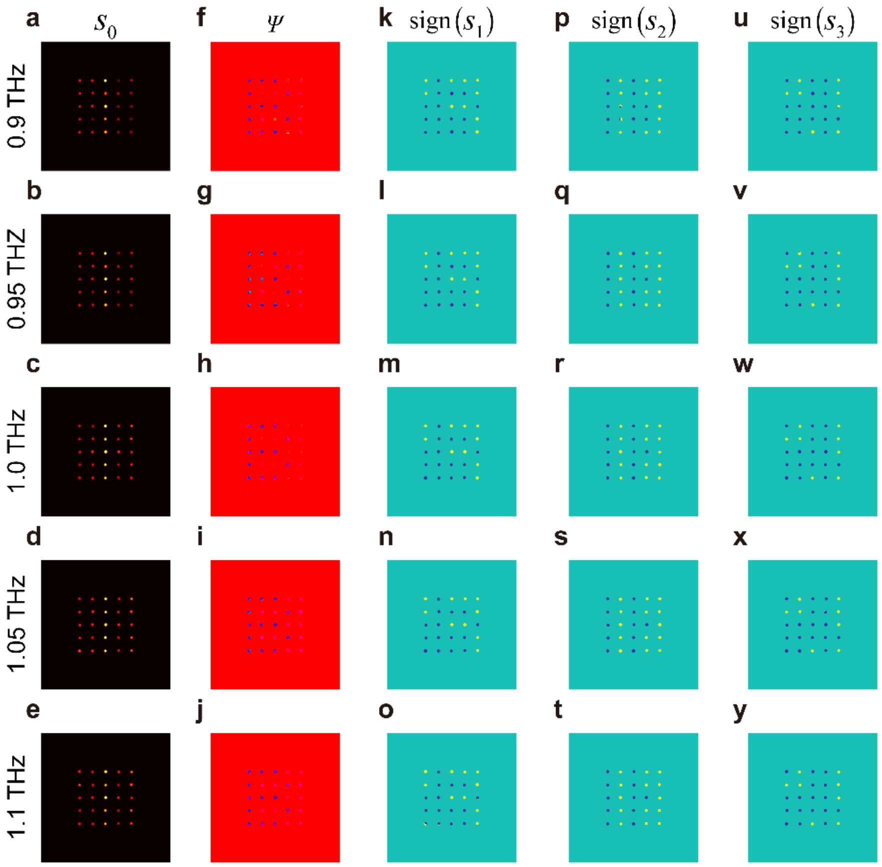

Figure S7. Simulation results of the information encryption meta-hologram at different frequencies. Simulated distributions of the holographic image in the a-e) s_0, f-j) Ψ, k-o) s_1, p-t) s_2 and u-y) s_3 channels from 0.9 to 1.1 THz with 0.05 THz steps, respectively. The working bandwidth is about 0.1 THz from 0.95 to 1.05 THz.

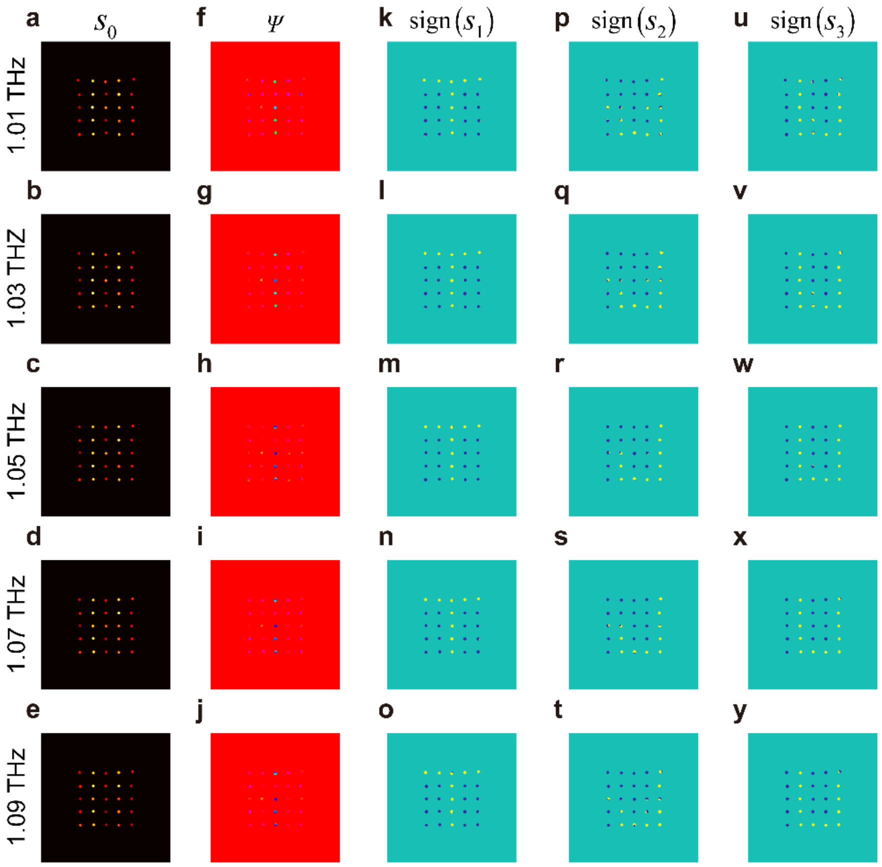

Figure S8. Measured results of the five-channel imaging meta-hologram at different frequencies. Measured distributions of the holographic image in the a-e) s_0, f-j) Ψ, k-o) s_1, p-t) s_2 and u-y) s_3 channels from 1.01 to 1.09 THz with 0.02 THz steps, respectively. The working bandwidth is about 0.02 THz from 1.05 to 1.07 THz.

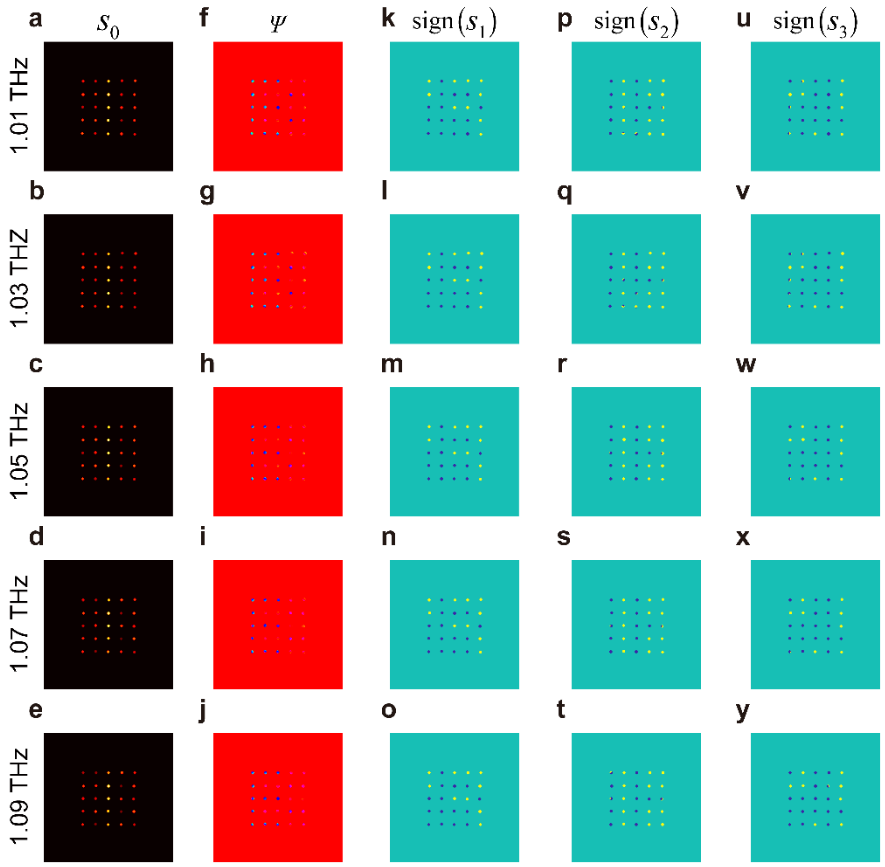

Figure S9. Measured results of the information encryption meta-hologram at different frequencies. Measured distributions of the holographic image in the a-e) s_0, f-j) Ψ, k-o) s_1, p-t) s_2 and u-y) s_3 channels from 1.01 to 1.09 THz with 0.02 THz steps, respectively. The effective working bandwidth is about 0.02 THz from 1.05 to 1.07 THz.

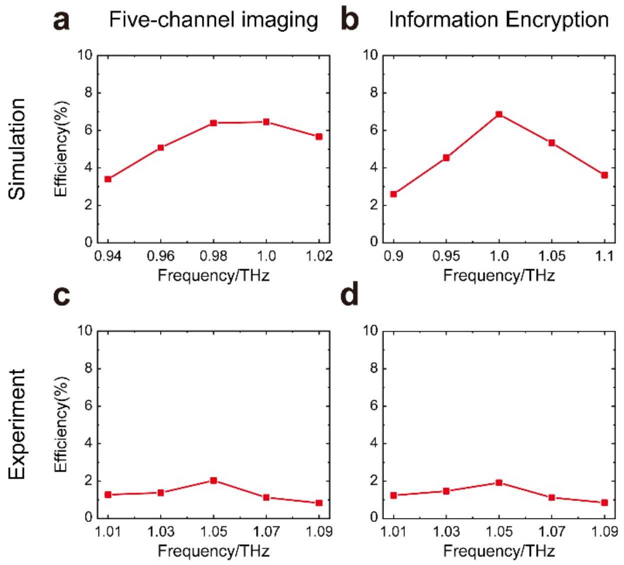

Figure S10. Efficiencies of the meta-holograms. Simulated a,b) and measured c,d) efficiencies of the five-channel imaging a,c) and information encryption b,d) meta-holograms at different frequencies, respectively.

Table S1. Codified cipher book 1.

BIN	Char	BIN	Char	BIN	Char	BIN	Char
000000	**STX**	010000	**c**	100000	**s**	110000	**-**
000001	**NL**	010001	**d**	100001	**t**	110001	*****
000010	**SPACE**	010010	**e**	100010	**u**	110010	**/**
000011	**NLC**	010011	**f**	100011	**v**	110011	**(**
000100	**0**	010100	**g**	100100	**w**	110100	**)**
000101	**1**	010101	**h**	100101	**x**	110101	**@**
000110	**2**	010110	**i**	100110	**y**	110110	**#**
000111	**3**	010111	**j**	100111	**z**	110111	**¥**
001000	**4**	011000	**k**	101000	**:**	111000	**%**
001001	**5**	011001	**l**	101001	**;**	111001	**^**
001010	**6**	011010	**m**	101010	**"**	111010	**&**
001011	**7**	011011	**n**	101011	**,**	111011	**|**
001100	**8**	011100	**o**	101100	**.**	111100	**~**
001101	**9**	011101	**p**	101101	**!**	111101	**>**
001110	**a**	011110	**q**	101110	**?**	111110	**<**
001111	**b**	011111	**r**	101111	**+**	111111	**ETX**

Table S2. Codified cipher book 2.

BIN	Char	BIN	Char	BIN	Char	BIN	Char
000000	**2**	010000	**x**	100000	**u**	110000	**w**
000001	**r**	010001	**s**	100001	**&**	110001	**7**
000010	**d**	010010	**:**	100010	**(**	110010	**NL**
000011	**"**	010011	**i**	100011	*****	110011	**#**
000100	**v**	010100	**l**	100100	**%**	110100	**-**
000101	**)**	010101	**SPACE**	100101	**/**	110101	**+**
000110	**o**	010110	**@**	100110	**;**	110110	**4**
000111	**y**	010111	**6**	100111	**e**	110111	**!**
001000	**ETX**	011000	**3**	101000	**<**	111000	**b**
001001	**^**	011001	**q**	101001	**~**	111001	**f**
001010	**?**	011010	**c**	101010	**,**	111010	**n**
001011	**¥**	011011	**>**	101011	**j**	111011	**NLC**
001100	**p**	011100	**8**	101100	**a**	111100	**z**
001101	**STX**	011101	**0**	101101	**5**	111101	**g**
001110	**|**	011110	**m**	101110	**9**	111110	**t**
001111	**1**	011111	**h**	101111	**.**	111111	**k**

References

[S1] P. Schober, C. Boer, L.A. Schwarte, *Anesth. Analg.* **2018,** 126, 1763.
